#  Percepção de imagem corporal, características socioeconômicas e estilo de vida em mulheres participantes do ELSA-Brasil na Bahia, Brasil 

**DOI:** 10.1590/0102-311XPT107823

**Published:** 2024-02-19

**Authors:** Ester Maria Dias Fernandes de Novaes, Estela M. L. Aquino, Ligia Gabrielli, Sheila Maria Alvim de Matos, Rosane Harter Griep, Maria de Jesus Mendes da Fonseca, Maria da Conceição Chagas de Almeida, Ana Luísa Patrão

**Affiliations:** 1 Instituto de Saúde Coletiva, Universidade Federal da Bahia, Salvador, Brasil.; 2 Instituto Oswaldo Cruz, Fundação Oswaldo Cruz, Rio de Janeiro, Brasil.; 3 Escola Nacional de Saúde Pública Sergio Arouca, Fundação Oswaldo Cruz, Rio de Janeiro, Brasil.; 4 Instituto Gonçalo Moniz, Fundação Oswaldo Cruz, Salvador, Brasil.; 5 Faculdade de Psicologia e de Ciências da Educação, Universidade do Porto, Porto, Portugal.

**Keywords:** Imagem Corporal, Identidade de Gênero, Fatores Socioeconômicos, Estilo de Vida, Comportamentos Relacionados com a Saúde, Body Image, Gender Identity, Socioeconomic Factors, Life Style, Health Behavior, Imagen Corporal, Identidad de Género, Factores Socioeconómicos, Estilo de Vida, Conductas Relacionadas con la Salud

## Abstract

Distorção da imagem corporal é uma alteração da percepção do corpo que pode repercutir na saúde. Este estudo visa estimar, entre mulheres participantes do *Estudo Longitudinal de Saúde do Adulto* (ELSA-Brasil) residentes na Bahia, Brasil, a prevalência de acurácia e distorção da imagem corporal e investigar associações com características socioeconômicas, estilo de vida e procura de cuidados ginecológicos. Participaram 609 mulheres de 50-69 anos de idade que responderam, entre 2012-2014, questionários aplicados face a face. Foi utilizada a escala de silhuetas de Stunkard para investigar a percepção acurada ou distorcida para mais ou menos peso. A razão de risco relativo (RR) foi calculada por meio de regressão logística multinomial por meio do Stata 13. A maioria das participantes tem perspectiva acurada do próprio corpo (53,7%). Entre aquelas com percepção distorcida, há uma tendência à distorção para menos peso (38,1%). Na análise de regressão multinomial, permaneceram associadas à distorção para menos peso as variáveis raça/cor e escolaridade, sendo que a primeira foi positivamente associada à distorção para menos peso entre as pardas (RR = 1,89; IC95%: 1,13-3,16) e pretas (RR = 2,10; IC95%: 1,25-3,55), enquanto a segunda entre aquelas com escolaridade até o Ensino Médio (RR = 1,65; IC95%: 1,18-2,33). Não houve associações quanto às demais variáveis, nem com distorção para mais peso. Os resultados contribuem para a explicação das relações entre percepção da imagem corporal e fatores socioeconômicos, revelando que mulheres de raça/cor diferentes e variados níveis de escolaridade são influenciadas de formas distintas pelos discursos sociais, o que impacta a percepção da sua imagem corporal.

## Abstract (en)

Body image distortion is an alteration in the perception of the body that can have repercussions on health. This study aims to estimate the prevalence of body image accuracy and distortion among women participating in the *Brazilian Longitudinal Study of Adult Health* (ELSA-Brasil) living in Bahia State, Brazil, and to investigate associations with socioeconomic characteristics, lifestyle, and gynecological care seeking. A total of 609 women aged 50 to 69 years participated in the study, who answered face-to-face questionnaires from 2012 to 2014. The Stunkard silhouette scale was used to investigate accurate or distorted perception for more or less weight. The relative risk ratio (RR) was calculated by multinomial logistic regression using Stata 13. Most participants have an accurate perception of their own bodies (53.7%). Among those with distorted perception, there is a tendency to distort towards less weight (38.1%). In the multinomial regression analysis, the variables race/skin color and education remained associated with the distortion towards underweight. The race/skin color variable was positively associated with the distortion towards underweight among Mixed-race women (RR = 1.89; 95%CI: 1.13-3.16) and black (RR = 2.10; 95%CI: 1.25-3.55), while the education variable among those with up to high school education (RR = 1.65; 95%CI: 1.18-2.33). There were no associations with the other variables or with distortion for more weight. The results contribute to explaining the relationships between body image perception and socioeconomic factors, revealing that women of different races/skin colors and varying educational levels are influenced in different ways by social discourses, impacting the perception of their body image.

## Abstract (es)

La distorsión de la imagen corporal es una alteración en la percepción del cuerpo que puede repercutir en la salud. Este estudio busca estimar, entre las mujeres participantes del *Estudio Longitudinal de Salud del Adulto* (ELSA-Brasil) que viven en Bahía, Brasil, la prevalencia de precisión y distorsión de la imagen corporal e investigar asociaciones con las características socioeconómicas, el estilo de vida y la busca de atención ginecológica. Participaron 609 mujeres que tenían entre 50 y 69 años que contestaron los cuestionarios aplicados cara a cara entre 2012 y 2014. Se utilizó la escala de siluetas de Stunkard para investigar la percepción precisa o distorsionada para más o menos peso. El cociente de riesgo relativo (RR) se calculó a través de regresión logística multinomial utilizando el Stata 13. La mayoría de los participantes tiene una perspectiva precisa del propio cuerpo (53,7%). Entre las personas con percepción distorsionada hay una tendencia a la distorsión para menos peso (38,1%). En el análisis de regresión multinomial, las variables raza/color y escolaridad permanecieron asociadas con la distorsión para menos peso, siendo la primera positivamente asociada con la distorsión para menos peso entre las mujeres pardas (RR = 1,89; IC95%: 1,13-3,16) y negras (RR = 2,10; IC95%: 1,25-3,55), mientras la segunda entre las mujeres que estudiaron hasta la enseñanza secundaria (RR = 1,65; IC95%: 1,18-2,33). No hubo asociaciones con las otras variables ni con la distorsión para más peso. Los resultados contribuyen para explicar las relaciones entre la percepción de la imagen corporal y los factores socioeconómicos, demostrando que mujeres de diferentes razas/colores y diferentes niveles de educación se influyen de distintas formas a través de discursos sociales, lo que impacta en la percepción de su imagen corporal.

## Introdução

Imagem corporal é um construto multidimensional que engloba autopercepções e atitudes em relação à aparência física de uma pessoa, sendo importante para a compreensão de questões fundamentais de identidade ^1^. Ela não está necessariamente correlacionada à aparência física exata, mas, para seu entendimento, são fundamentais as atitudes e avaliações que o indivíduo faz de seu corpo ^2^.

A imagem corporal não é estática, a cultura interfere na sua formulação ^3^ e influencia a relação que o indivíduo estabelece com o próprio corpo. Ela é composta por diferentes elementos, dos quais a percepção é fator preponderante para compreender as representações subjetivas do corpo ^4^. Isso ocorre porque ela não só revela a forma como o sujeito se percebe, mas também manifesta se essa percepção é acurada ou distorcida - se há precisão ou diferença, por exemplo, entre o tamanho do corpo e a noção que se tem dele ^5^. Assim, uma percepção distorcida, seja para mais ou menos peso, favorece o surgimento de inúmeros problemas, podendo afetar a saúde e influenciar a autoestima, o humor, a competência, o funcionamento social e ocupacional ^6^.

Quando investigada por meio de uma perspectiva de gênero, os efeitos da distorção da imagem corporal se intensificam, pois o gênero é um determinante social da saúde que se relaciona com oportunidades e riscos ^7^. Como as mulheres costumam experienciar uma pressão social sobre a sua aparência diferente daquela vivida pelos homens ^1^, não se pode desconsiderar essa relação, nem deixar de analisar outros fatores socioeconômicos, como raça/cor ^8^^,^^9^^,^^10^, classe social e escolaridade ^8^^,^^11^. Aqui, por exemplo, destaca-se que esses fatores, além de situarem questões individuais, também favorecem opressões sociais cruzadas que se relacionam diretamente com a saúde ^12^. Isto é, questões como classe social e raça se associam ao gênero e têm efeitos nos comportamentos de saúde, operando de forma interseccional e não meramente aditiva ^12^, fazendo com que essas populações experimentem variados níveis de desigualdade.

Semelhantemente, estudos recentes revelam que a distorção da imagem corporal pode se associar ao estilo de vida, contribuindo para gerar mudanças comportamentais relacionadas à saúde física, como alterações nutricionais ^5^^,^^13^, atividade física ^14^, consumo excessivo de álcool ^15^, e à saúde mental ^13^, influenciando a procura por cuidados de saúde ^16^. Essa procura se traduz como aspecto fundamental, já que a distorção da imagem corporal pode favorecer menor engajamento em comportamentos relacionados à saúde e apresentar maiores riscos para ela ^17^.

Em estudo realizado por DeMaria et al. ^18^, que buscou avaliar se a autoimagem genital, a imagem corporal e os comportamentos sexuais predizem comportamentos de exame ginecológico em uma amostra de mulheres universitárias, foi identificado que, embora a autoimagem genital e a imagem corporal não tenham emergido como os únicos preditores mais fortes de comportamentos do exame ginecológico, seu impacto não deve ser desconsiderado nem passar despercebido, pois é necessário investigar de forma mais assertiva os motivos que auxiliam na tomada de decisão da mulher quanto à ação de buscar cuidados ginecológicos. Por essa razão, é importante e recomendado que haja a promoção de uma imagem corporal positiva, a fim de possibilitar uma melhor qualidade de vida, melhor saúde física e auxiliar em melhores escolhas dos comportamentos relacionados à saúde ^1^. Nesse sentido, infere-se que uma imagem corporal mais acurada pode favorecer a adoção de um estilo de vida mais saudável e também repercutir sobre uma maior procura por cuidados em saúde. É o que constatam Patrão et al. ^19^ ao concluírem que mulheres menos comprometidas com a realização de exames mamográficos foram as que mais consumiram álcool em excesso e eram menos ativas fisicamente, o que sugeriu que a realização da mamografia e hábitos de vida saudáveis estão interligados. Ou seja, identificou-se correlação entre comportamentos de risco, nesse caso, o consumo de álcool, e intervalo maior que o recomendado entre as mamografias.

Diante do exposto, este estudo tem o objetivo de estimar a prevalência de acurácia e distorção da imagem corporal e suas associações com características socioeconômicas, estilo de vida e procura de cuidados de saúde ginecológica entre mulheres residentes na Bahia, Brasil, participantes do *Estudo Longitudinal da Saúde do Adulto* (ELSA-Brasil).

## Materiais e métodos

### Desenho de estudo e população

Este estudo integra a pesquisa *Cuidados à Saúde, Fatores de Risco Clássicos e Detecção Precoce do Câncer de Mama entre Trabalhadoras de Universidade Pública da Bahia*, investigação realizada em 2014, suplementar ao ELSA-Brasil (maior coorte sobre saúde do adulto na América do Sul, cujos detalhes estão publicados em Aquino et al. ^20^), que tem como objetivo investigar o padrão de utilização dos serviços de saúde públicos e privados para consultas ginecológicas, exames clínicos e de imagem da mama.

Os dados analisados partiram inicialmente do total de 770 mulheres participantes. Foram elegíveis para a entrevista mulheres ativas e aposentadas, com idade entre 50-69 anos, que não apresentavam problemas cognitivos e/ou deficiências físicas que as impedissem de conceder informações e que não realizaram mamografia no último ano. Esses critérios foram utilizados considerando a prerrogativa do Ministério da Saúde, que institui a única estratégia brasileira voltada para o rastreamento mamográfico, determinando que seja realizado em período bienal entre mulheres de 50-69 anos ^21^.

Foram excluídas aquelas que estavam muito doentes ou haviam se mudado de Salvador; que não informaram sobre o tempo desde a realização do último exame mamográfico; que se autodeclararam como raça/cor amarela (de origem asiática) e indígena (devido ao pequeno número e especificidades que dificultam agregá-las a outra categoria); que não responderam à questão sobre raça/cor; e que não responderam à pergunta sobre percepção da imagem corporal. Com essas exclusões, foram efetivamente analisadas 609 mulheres.

### Instrumentos e variáveis

Utilizaram-se informações sobre a percepção da imagem corporal, características socioeconômicas e estilo de vida incluídas no questionário aplicado no segundo seguimento (2012-2014) do ELSA-Brasil, além de informações sobre cuidados ginecológicos, local de nascimento e de residência das participantes obtidos em estudo suplementar ^22^, que realizou adaptação do questionário do Projeto de Agrupamento Internacional sobre Densidade Mamográfica (*International Pooling Project on Mammographic Density*). As variáveis foram mensuradas conforme a seguir.

#### • Variável dependente

Percepção da imagem corporal: medida segundo a acurácia e distorção por meio dos resultados da aplicação da escala de silhueta de Stunkard ^23^, que consiste em nove figuras de silhuetas que variam gradualmente de tamanho (de muito magras a muito obesas). Seguindo a estratificação adotada por outros autores ^24^, as referidas figuras foram classificadas em baixo peso, peso normal, sobrepeso e obesidade, sendo cada imagem representada por um intervalo de índice de massa corporal (IMC, em kg/m^2^) a partir desse parâmetro, a saber: baixo peso (IMC < 18,5), peso normal (IMC entre 18,5-24,9), sobrepeso (IMC entre 25,0-29,9) e obesidade (IMC > 30,0). Foi considerado como resposta o número da figura selecionada pelas participantes diante da questão “escolha a figura que reflete como você pensa que se parece”. As participantes tiveram o seu IMC calculado a partir da obtenção de suas medidas de peso em quilogramas (aferido em balança digital portátil) e estatura em metros (aferida em estadiômetro portátil) e realizado o cálculo padrão de peso ÷ (altura × altura). A percepção da imagem corporal foi considerada a partir da acurácia ou distorção entre esse cálculo e a figura selecionada, sendo -1/+1 o ponto de corte. A discrepância da percepção da imagem corporal correspondeu à subtração da percepção da imagem corporal e o IMC. Ou seja, a escolha de uma figura menor do que o IMC correspondente ao próprio peso e altura foi caracterizada como distorção para menos peso, enquanto a escolha de uma figura maior foi considerada distorção para mais peso. Por último, a escolha da figura correspondente à sua faixa de IMC caracterizou a acurácia. Assim, a variável foi categorizada em percepção acurada, distorção para menos peso e distorção para mais peso.

#### • Variáveis independentes (ou explicativas)

Socioeconômicas - cinco variáveis avaliaram fatores sociais e econômicos. São elas:

(a) Raça/cor: autodesignação de acordo com os critérios do Instituto Brasileiro de Geografia e Estatística (IBGE), que classifica a raça/cor em preta, parda, branca, amarela ou indígena;

(b) Idade: questão sobre a data de nascimento da participante, sendo categorizada entre 50-59 anos e 60-69 anos;

(c) Classe social: a partir das categorias baixa, média ou alta - indicador baseado no tipo de trabalho que o indivíduo realiza, nas funções que exerce e no nível de escolaridade, categorizado segundo a Classificação Brasileira de Ocupações ^25^;

(d) Nível de escolaridade: resposta à pergunta “qual é seu grau de instrução?”, tendo como alternativas de respostas as categorias: 1º grau incompleto, 1º grau completo, 2º grau incompleto, 2º grau completo, universitário incompleto, universitário completo ou pós-graduação. Essa variável foi categorizada em até Ensino Médio e Ensino Superior;

(e) Local de nascimento e de residência: obtida por meio de perguntas sobre município de origem e atual da participante, se sede ou zona rural, tempo de residência e porte da cidade. As categorias foram separadas entre nascidos e residentes no interior e capital.

Estilo de vida - em estudo anterior, Patrão et al. ^26^ selecionaram quatro variáveis relativas ao estilo de vida. Optou-se por utilizá-las nesta investigação e também por acrescentar a variável sono. As variáveis selecionadas são:

(a) Dieta: autorrelato sobre a mudança de hábitos alimentares ou realização de regime alimentar nos últimos seis meses, sendo categorizada como “sim” aquelas que afirmam praticar a dieta e “não” aquelas que não o fazem.

(b) Prática de atividade física: utilizando a versão brasileira, validada por Matsudo et al. ^27^, do *Questionário Internacional de Atividade Física*^28^, as participantes foram classificadas em ativas (aquelas que relataram ≥ 150 minutos de caminhada ou atividade física moderada por semana ou ≥ 60 minutos de atividade física extenuante por semana) e insuficientemente ativas (aquelas que relataram < 150 minutos de caminhada ou atividade física moderada por semana ou < 60 minutos de extenuante atividade física por semana);

(c) Consumo excessivo de álcool: questões sobre ingestão semanal de bebidas alcoólicas (vinho tinto e branco, chope, cerveja de garrafa e destilados) e classificada como excessivo ou não (< 140g/semana = não excessivo; ≥ 140g/semana = excessivo), seguindo os parâmetros adotados por Duncan et al. ^29^, a exemplo da pergunta “quantas taças de vinho tinto a senhora consome por semana?”;

(d) Tabagismo: por meio da pergunta “a senhora fuma cigarros atualmente?”, as participantes foram categorizadas entre nunca fumou e já fumou ou fuma atualmente;

(e) Sono: quantidade de horas de sono relatada, em resposta à pergunta “quantas horas, em média, a senhora dorme numa noite habitual de sono?”. As respostas foram classificadas entre < 7 horas e ≥ 7 horas ^30^.

Cuidados com a saúde ginecológica - foram selecionadas cinco variáveis, as quais foram categorizadas conforme preconização do Ministério da Saúde ^31^^,^^32^, para os cuidados mamográficos e ginecológicos. São elas:

(a) Periodicidade do exame clínico das mamas: quando foi a última vez que um médico ou enfermeira fez seu exame clínico das mamas, com respostas variando entre < 2 anos e 2 anos ou mais;

(b) Periodicidade da mamografia: tempo desde a realização do exame de imagem, periodicidade e motivo da não realização, por meio da pergunta “quando foi a última vez que a senhora fez um exame de mamografia?”. As respostas variavam entre < 2 anos e 2 anos ou mais;

(c) Periodicidade da consulta ginecológica: pergunta que investiga quando foi a última vez que a participante fez uma consulta ginecológica, com resposta variando entre < 3 anos e 3 anos ou mais;

(d) Periodicidade de exame citológico: em resposta à pergunta “qual foi a última vez que a senhora fez um exame preventivo para câncer de colo do útero?”, cujas respostas variavam entre < 3 anos e 3 anos ou mais;

(e) Uso de terapia de reposição hormonal: uso de medicamentos com hormônios para a menopausa e o tempo de uso, por meio da da pergunta: “a senhora usa ou já usou hormônios para tratamento de sintomas da menopausa?”. As respostas variaram entre sim e não.

### Análise de dados

A distribuição de frequência das variáveis independentes (socioeconômicas, estilo de vida e cuidados com a saúde ginecológica) foi calculada segundo a percepção da imagem corporal. A percepção acurada/distorcida foi o desfecho e as variáveis socioeconômicas, comportamentos relacionados ao estilo de vida e procura por cuidados ginecológicos constituíram os fatores de exposição. “Percepção acurada” foi considerada a categoria de referência na análise. As medidas de associação foram calculadas utilizando regressão logística multinomial. Selecionaram-se as covariáveis com valores de p < 0,20 na análise estratificada (Tabela 1) para inclusão nos modelos finais (Tabela 2), os quais foram devidamente ajustados. Essa seleção utilizou critério menos conservador a partir dos resultados do qui-quadrado (Tabela 1) para incluir mais variáveis no modelo com base na literatura e atendeu a critérios teóricos e estatísticos. As razões de risco relativo (RR), medidas escolhidas pelo uso de variável politômica e seus intervalos de 95% de confiança (IC95%) foram calculados como medidas de associação. Utilizou-se o software estatístico Stata, versão 13 (https://www.stata.com).

**Tabela 1:** Características socioeconômicas, relacionadas ao estilo de vida e aos cuidados ginecológicos das participantes do ELSA-Brasil (*Estudo Longitudinal da Saúde do Adulto*), residentes na Bahia, Brasil, de acordo com a percepção de sua imagem corporal (2012-2014).

Características	Distorção da imagem corporal						Valor de p *
	Percepção acurada [n = 327]		Distorção para mais peso [n = 50]		Distorção para menos peso [n = 232]		
	n	%	n	%	n	%	
Fatores socioeconômicos							
Faixa etária (anos)							0,022
50-59	192	55,2	36	10,3	120	34,5	
60-69	135	51,7	14	5,4	112	42,9	
Raça/Cor autorreferida							0,077
Branca	68	66,0	8	7,8	27	26,2	
Parda	136	52,3	22	8,5	102	39,2	
Preta	117	50,0	19	8,1	98	41,9	
Classe social							0,443
Alta	88	58,3	15	9,9	48	31,8	
Média	175	52,9	26	7,8	130	39,3	
Baixa	63	50,8	9	7,3	52	41,9	
Escolaridade							0,013
Ensino Superior	169	59,3	25	8,8	91	31,9	
Até Ensino Médio	158	48,8	25	7,7	141	43,5	
Local de nascimento							0,122
Interior	120	55,6	23	10,6	73	33,8	
Capital	205	52,6	27	6,9	158	40,5	
Local de residência							0,409
Interior	22	64,7	2	5,9	10	29,4	
Capital	304	53,0	48	8,3	222	38,7	
Fatores relacionados ao estilo de vida							
Faz dieta							0,030
Não	204	54,7	38	10,2	131	35,1	
Sim	123	52,1	12	5,1	101	42,8	
Atividade física							0,617
Ativo	108	56,5	14	7,4	69	36,1	
Insuficientemente ativo	219	52,4	36	8,6	163	39,0	
Consumo excessivo de álcool							0,192
Não	307	53,8	44	7,7	220	38,5	
Sim	20	52,6	6	15,8	12	31,6	
Tabagismo							0,122
Nunca fumou	208	51,6	39	9,7	156	38,7	
Já fumou ou fuma atualmente	119	57,8	11	5,3	76	36,9	
Sono (horas)							0,834
< 7	187	54,0	30	8,7	129	37,3	
≥ 7	140	53,2	20	7,6	103	39,2	
Fatores relativos aos cuidados ginecológicos							
Periodicidade do exame clínico das mamas (anos)							0,824
< 2	263	52,9	43	8,7	191	38,4	
≥ 2	56	54,9	7	6,9	39	38,2	
Periodicidade da mamografia (anos)							0,462
< 2	267	54,6	38	7,8	184	37,6	
≥ 2	56	49,1	12	10,5	46	40,4	
Periodicidade da consulta ginecológica (anos)							0,177
< 3	299	52,8	49	8,7	218	38,5	
≥ 3	27	65,9	1	2,4	13	31,7	
Periodicidade do exame citológico (anos)							0,882
< 3	286	53,2	45	8,3	207	38,5	
≥ 3	32	56,1	5	8,8	20	35,1	
Já usou terapia hormonal							0,823
Não	157	51,5	22	7,2	126	41,3	
Sim	83	53,9	12	7,8	59	38,3	
Usa terapia hormonal							0,141
Não	239	52,3	33	7,2	185	40,5	
Sim	62	57,4	12	11,1	34	31,5	

* Em negrito: p < 0,20.

### Considerações éticas

Esta pesquisa seguiu as Resoluções do Conselho Nacional de Saúde *nº 466/2012* e *nº 510/2016* e demais resoluções internacionais. O protocolo ELSA-Brasil foi submetido e aprovado pelos Comitês de Ética em Pesquisa de cada instituição participante e pela Comissão Nacional de Ética em Pesquisa, tendo os seguintes CAAE: Instituto de Saúde Coletiva da Universidade Federal da Bahia (0017.1.069.000-06), Fundação Oswaldo Cruz (0058.0.011.000-07), Hospital Universitário da Universidade de São Paulo (0016.1.198.000-06), Universidade Federal de Minas Gerais (0186.1.203.000-06), Centro de Ciências da Saúde da Universidade Federal do Espírito Santo (08109612.7.2003.5060) e Hospital de Clínicas de Porto Alegre (48608515.5.1001.5327).

## Resultados

**Figura 1:**
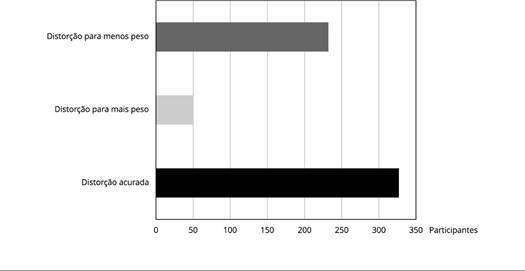
Distribuição das participantes do ELSA-Brasil (*Estudo Longitudinal da Saúde do Adulto*), residentes na Bahia, Brasil, segundo percepção da imagem corporal (2012-2014).

Foram incluídas 609 mulheres neste estudo, dentre as quais 53,7% tinham a percepção da imagem corporal acurada. As demais apresentaram imagem distorcida: 38,1% se veem com menor e 8,2% com maior massa corporal do que o efetivamente calculado (Figura 1).

As participantes com faixa etária entre 50-59 anos tinham percepção mais acurada sobre a imagem corporal do que aquelas entre 60-69 anos. Apesar disso, quando comparados os grupos, as mulheres mais jovens apresentaram maior distorção para mais peso do que as mais velhas, que, por sua vez, demonstraram distorção mais prevalente para menos peso (Tabela 1).

As mulheres autorreferidas brancas mostraram percepção mais acurada da imagem corporal quando comparadas às pardas e, principalmente, às pretas. Estas últimas, aliás, apresentaram a maior distorção para menos peso entre os grupos analisados (Tabela 1).

Mulheres com Ensino Superior completo apresentaram percepção mais acurada e as que estudaram até o Ensino Médio apresentaram maior distorção para menos peso (Tabela 1). Acerca dos fatores relativos ao estilo de vida, constatou-se associação significante com a dieta - tanto entre as mulheres que não faziam dieta quanto entre as que faziam, mais da metade tinham percepção acurada; todavia, estas últimas revelaram maior distorção para menos peso se comparadas ao primeiro grupo. Associações com as demais características socioeconômicas não foram estatisticamente significantes, assim como ocorreu com os cuidados ginecológicos (Tabela 1).

Na análise de regressão multinomial, permaneceram associadas à distorção da imagem corporal as variáveis raça/cor autorreferida e escolaridade, sendo que a primeira foi positivamente associada à distorção para menos peso entre as pardas (RR = 1,89; IC95%: 1,13-3,16) e pretas (RR = 2,10; IC95%: 1,25-3,55), enquanto a segunda foi associada entre aquelas com escolaridade até o Ensino Médio (RR = 1,65; IC95%: 1,18-2,33) (Tabela 2).

Não foram encontradas associações estatisticamente significantes entre as variáveis selecionadas e a distorção para mais peso (Tabela 2).

**Tabela 2:** Associações entre as características selecionadas e a distorção da imagem corporal das participantes do ELSA-Brasil (*Estudo Longitudinal da Saúde do Adulto*), residentes na Bahia, Brasil (2012-2014).

Variáveis	Distorção para menos peso		Distorção para mais peso	
	RR	IC95%	RR	IC95%
Faixa etária (anos)				
50-59	1,00		1,00	
60-69	1,33	0,94-1,86	0,55	0,29-1,06
Raça/Cor autorreferida				
Branca	1,00		1,00	
Parda	1,89	1,13-3,16	1,37	0,58-3,25
Preta	2,10	1,25-3,55	1,38	0,57-3,32
Escolaridade				
Ensino Superior	1,00		1,00	
Até Ensino Médio	1,65	1,18-2,33	1,07	0,59-1,94
Local de nascimento				
Interior	1,00		1,00	
Capital	1,27	0,89-1,81	0,69	0,38-1,25
Dieta				
Não	1,00		1,00	
Sim	1,28	0,91-1,80	0,52	0,26-1,04
Uso excessivo de álcool				
Não	1,00		1,00	
Sim	0,84	0,40-1,75	2,09	0,80-5,50
Tabagismo				
Nunca fumou	1,00		1,00	
Já fumou ou fuma atualmente	0,85	0,60-1,21	0,49	0,24-1,00
Periodicidade da consulta ginecológica (anos)				
< 3	1,00		1,00	
≥ 3	0,66	0,33-1,31	0,23	0,30-1,70
Uso atual de terapia hormonal				
Não	1,00		1,00	
Sim	0,71	0,45-1,12	1,40	0,68-2,87

IC95%: intervalo de 95% de confiança; RR: razão de risco relativo.

Nota: modelo ajustado.

## Discussão

Este estudo mostrou que pouco mais da metade das participantes apresentou percepção acurada da imagem corporal. Esse achado é interessante porque, embora o ponto de corte adotado possa ser entendido como pouco flexível quanto à avaliação da percepção da própria imagem corporal, ele mostra que um percentual expressivo das participantes conseguiu revelar acurácia quanto à sua imagem percebida, o que também pode apontar para a sensibilidade da escala adotada.

Constatou-se, ainda, que o perfil entre as mulheres que revelaram distorção para mais peso é de pessoas mais jovens, de raça/cor parda, com maior escolaridade e que não costumam fazer dieta. Já entre as que distorcem para menos peso, estão as mulheres mais velhas, de raça/cor preta, com menor escolaridade e que costumam fazer dieta.

Esses achados são semelhantes aos encontrados na literatura. Em pesquisa conduzida por Song et al. ^33^, 25,7% das mulheres de meia-idade apresentaram distorção da imagem corporal. Já em Pandolfi et al. ^5^, 75% das mulheres com idade entre 20-59 anos classificaram sua silhueta de forma menos acurada, superestimando o seu tamanho. Resultado semelhante foi encontrado por Bellard et al. ^34^, em que tanto as mulheres mais jovens quanto as de meia-idade preferiram um tamanho corporal mais magro quando solicitadas a julgar como gostariam de aparecer, superestimando seu tamanho corporal atual percebido pelo aumento do IMC. Uma possível explicação é o que tem sido chamado, nas sociedades modernas, de “duplo padrão de envelhecimento”, que mostra dois padrões de beleza masculina (o menino e o homem), sendo possível, para a figura masculina, envelhecer e mostrar sinais físicos do amadurecimento, e apenas um padrão feminino (a menina), o que faz com que o processo de envelhecimento seja visto a partir de um viés negativo e problemático pelas mulheres ^35^, que precisam se manter eternamente jovens e magras.

No que diz respeito à raça/cor, os resultados revelaram que as mulheres brancas mostraram uma percepção mais acurada da imagem corporal do que as pardas e pretas, o que contraria dados que demonstraram que as mulheres brancas costumam ter uma percepção mais distorcida, geralmente escolhendo um tamanho corporal ideal significativamente mais magro do que as mulheres negras e expressando maior preocupação com peso e dieta ^36^. Por outro lado, em estudo que também utilizou a base de dados do ELSA-Brasil, mas analisou as categorias satisfação/insatisfação, as mulheres negras apresentaram maiores chances de insatisfação leve e moderada devido à magreza ^37^, indicando diferenças culturais relevantes quanto à imagem ideal de corpo, que pode ter seu sentido mais voltado para trabalho e reprodução.

Isso permite refletir sobre como o processo de leitura da imagem corporal atravessa de forma variada as diferentes mulheres. Conforme esclarece Banks ^38^, ao longo da história, as mulheres negras tiveram seus corpos e beleza desvalorizados pela cultura dominante em detrimento de uma supervalorização da estética europeia. Com isso, o modelo de beleza difundido buscava padronizar os corpos de modo a tornar imperativo que, para ser bonita, era preciso seguir o ideal europeu, o que aumenta a distorção e a insatisfação com a imagem corporal. No entanto, devido aos ideais culturais que promovem um tipo de corpo com medidas distintas daquelas geralmente difundidas em massa, as mulheres negras podem ter se tornado menos suscetíveis à insatisfação corporal do que as brancas ^36^, embora não estejam livres dessa influência. Isso pode explicar, por exemplo, porque as mulheres negras deste estudo apresentaram distorção para menos peso sobre sua imagem corporal.

Quanto à escolaridade, as mulheres que distorcem para mais peso têm maior escolaridade e as que distorcem para menos peso são menos escolarizadas. A literatura mostra que, entre as participantes do ELSA-Brasil, as chances de subestimar o tamanho corporal aumentaram três vezes em relação à menor escolaridade, enquanto a escolaridade primária e a baixa renda *per capita* diminuíram em 30% as chances de superestimar ligeiramente o tamanho corporal ^37^. Ou seja, a escolaridade demonstra relação importante com a percepção da imagem corporal entre as mulheres, de forma que, quanto menor a escolaridade, mais elas subestimam o próprio tamanho. Isso ganha importância na medida em que pode influenciar a adoção de comportamentos de saúde de risco, uma vez que essas mulheres, por não terem uma percepção precisa de sua imagem corporal, podem adotar hábitos que prejudiquem a saúde ^17^.

Este estudo verificou que mulheres que estudaram até o Ensino Médio também apresentaram distorção da imagem corporal para menos peso. Entre os autores que estudaram a relação dessa variável com a insatisfação da imagem corporal, Carvalho et al. ^39^, ao analisarem imagem corporal em mulheres idosas e fatores associados, não encontraram relação com a escolaridade. Em contrapartida, Marques et al. ^40^ demonstraram que os resultados referentes ao nível educacional marcam maior insatisfação corporal entre as mulheres mais escolarizadas. Fonseca et al. ^37^, por sua vez, revelaram que tanto homens quanto mulheres com menor escolaridade apresentaram maiores chances de insatisfação devido ao excesso de peso. Essas diferentes perspectivas mostram as possibilidades de interação da variável escolaridade a partir da população estudada. Para os resultados aqui encontrados, em particular, reconhece-se que as participantes compõem um grupo mais homogêneo e, por vezes, medicalizado. Sendo assim, refletir sobre as representações que seus corpos têm para elas e os significados que lhes são atribuídos é necessário, sobretudo porque, à medida que as mulheres se percebem de maneira distorcida, a adoção de hábitos e comportamentos de risco pode impactar negativamente sua saúde ^41^. Portanto, as repercussões da percepção da imagem corporal precisam ser avaliadas porque os riscos associados à percepção incorreta do próprio peso podem ser danosos à saúde.

A literatura aponta que as mulheres que percebiam sua imagem corporal de forma distorcida estavam mais engajadas em comportamentos prejudiciais à saúde, como comportamentos alimentares desordenados ^41^, ingestão excessiva de álcool ^37^, não realização de atividades físicas, tabagismo e sono insuficiente ^17^. Neste estudo, a dieta apresentou associação com a distorção da imagem corporal. Poucos artigos que discutem essa associação foram encontrados; contudo, achados anteriores de pesquisas realizadas no ELSA-Brasil apresentaram resultados semelhantes quanto à relação da dieta com a insatisfação com a imagem corporal. Albuquerque et al. ^13^, por exemplo, encontraram que, entre as mulheres, havia chances maiores de insatisfação com a imagem corporal por baixo peso e um baixo consumo semanal de frutas, o que poderia estar relacionado à insatisfação observada. Além disso, Silva et al. ^42^ concluíram que a insatisfação com a imagem corporal pode influenciar os hábitos alimentares e de atividade física, determinando a percepção do seu estado geral de saúde. Isso aponta para a relevância de desenvolver estratégias que incentivem melhores hábitos alimentares na população.

Sobre a procura por cuidados de saúde ginecológica, não foram verificadas associações estatisticamente significantes com a percepção da imagem corporal, o que pode se dever ao baixo poder amostral. Nesse sentido, estudos futuros com populações maiores poderão ser elucidativos.

Diante desses achados, por meio do modelo multinomial, foram buscadas associações entre as variáveis identificadas como significativas na distribuição das frequências. Assim, enquanto o modelo de distorção para mais peso não manifestou associações com significância estatística, o modelo de distorção para menos peso revelou que duas características socioeconômicas apresentam tal associação: raça/cor e escolaridade.

Posto isso, cabe destacar que este estudo teve limitações. A primeira se refere ao tamanho da amostra analisada, uma vez que, por falta de poder amostral, não foi possível confirmar diferenças descritas na literatura quanto à relação de outras variáveis socioeconômicas, de estilo de vida e de saúde com a percepção da imagem corporal. Outro ponto é que ainda existem poucos estudos sobre percepção da imagem corporal, logo, embora a variável “satisfação” não tenha sido aqui analisada, seus estudos foram utilizados por terem sido desenvolvidos em maior escala. Todavia, como nem sempre a percepção acurada significa satisfação com a imagem corporal, estudos que analisem satisfação entre as participantes também podem ser pertinentes.

Quanto aos pontos fortes, vale salientar a importância deste trabalho para os estudos de gênero e percepção da imagem corporal, além de sua contribuição para o debate acerca da relação desta última com fatores socioeconômicos, sobretudo raça/cor e escolaridade. Por fim, destaca-se, também, que, apesar de não terem sido encontrados resultados estatisticamente significantes entre a procura de cuidados ginecológicos e a percepção da imagem corporal, esta investigação abre um campo de discussão para avaliar as motivações e empecilhos que auxiliam ou dificultam a busca de assistência à saúde por parte das mulheres, bem como promove maior atenção para o estudo da percepção da imagem corporal em associação aos cuidados em saúde.

## Conclusão

Os resultados deste estudo revelaram que a maioria das participantes tem perspectiva acurada do próprio corpo, enquanto, entre aquelas com percepção distorcida, há uma tendência à distorção para menos peso. Permaneceram associadas à distorção para menos peso as variáveis raça/cor e escolaridade, sendo que a primeira foi positivamente associada à distorção para menos peso entre as pardas e pretas, enquanto a segunda entre aquelas com escolaridade até o Ensino Médio.

Esses achados contribuem para a explicação das relações entre percepção da imagem corporal e fatores socioeconômicos, bem como apontam para a relevância de aspectos relacionados ao estilo de vida; afinal, eles demonstram que as mulheres não são afetadas pelos discursos sociais de forma homogênea: alguns grupos são atingidos mais diretamente do que outros e isso impacta seus modos de vida e a percepção da própria imagem corporal.
